# Adjusting the physico-chemical properties of collagen scaffolds to accommodate primary osteoblasts and endothelial cells

**DOI:** 10.1093/rb/rbad015

**Published:** 2023-03-10

**Authors:** Nima Meyer, Daniel V Bax, Jochen Beck, Ruth E Cameron, Serena M Best

**Affiliations:** Department of Materials Science and Metallurgy, University of Cambridge, Cambridge CB3 0FS, UK; Department of Materials Science and Metallurgy, University of Cambridge, Cambridge CB3 0FS, UK; Geistlich Pharma AG, Product Development, Wolhusen, Switzerland; Department of Materials Science and Metallurgy, University of Cambridge, Cambridge CB3 0FS, UK; Department of Materials Science and Metallurgy, University of Cambridge, Cambridge CB3 0FS, UK

**Keywords:** angiogenesis, collagen scaffolds, cell migration, EDC/NHS cross-linking

## Abstract

Collagen-based biomaterials are used widely as tissue engineering scaffolds because of their excellent bioactivity and their similarity to the natural ECM. The regeneration of healthy bone tissue requires simultaneous support for both osteoblasts and, where angiogenesis is intended, endothelial cells. Hence it is important to tailor carefully the biochemical and structural characteristics of the scaffold to suit the needs of each cell type. This work describes for the first time a systematic study to gain insight into the cell type-specific response of primary human osteoblast (hOBs) and human dermal microvascular endothelial cells (HDMECs) to insoluble collagen-based biomaterials. The behaviour was evaluated on both 2D films and 3D scaffolds, produced using freeze-drying. The collagen was cross-linked at various EDC/NHS concentrations and mono-cultured with hOBs and HDMECs to assess the effect of architectural features and scaffold stabilization on cell behaviour. It was observed that 3D scaffolds cross-linked at 30% of the standard conditions in literature offered an optimal combination of mechanical stiffness and cellular response for both cell types, although endothelial cells were more sensitive to the degree of cross-linking than hOBs. Architectural features have a time-dependent impact on the cell migration profile, with alignment being the most influential parameter overall.

## Graphical Abstract

**Figure:**
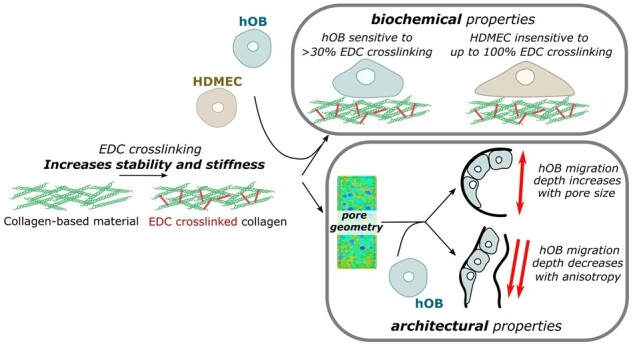

## Introduction

Bone tissue engineering offers a promising alternative to conventional bone grafting, overcoming disadvantages including limited availability and the need for double surgery. However, achieving the necessary complexity of bone tissue, containing both bone cells and an intricate blood vessel network remains challenging. To allow appropriate tissue regeneration a close resemblance of the natural process involving simultaneous growth of bone and blood vessels through angiogenesis is required. This process includes a complex successive cascade of very tightly regulated interactions between various growth factors, different cell types and the extracellular matrix (ECM) [1]. Improving the outcome of engineered tissue requires optimization of the synergy between these necessary signals, various cell types and the scaffold material which serves as a temporary substitute for the ECM.

The abundance of collagen in human tissue, mainly skin and bone, combined with its beneficial features such as cell recognition sites, tunable biodegradability, biocompatibility and availability make it an ideal candidate for tissue engineered scaffolds. Using freeze-drying, 3D collagen scaffolds can be produced with a tunable architecture allowing adjustment of structural features, such as pore size, interconnectivity, percolation diameter and scaffold stiffness. These parameters are of key importance as they not only influence cell migration into the scaffolds but also ensure cellular nutrition and transport of waste products. Understanding the interaction between a biomaterial and such a biological system will help to improve the scaffold’s functionality [2, 3].

Despite numerous advantages, as-prepared freeze-dried 3D collagen scaffolds possess relatively low structural stability and mechanical stiffness. To improve these properties, an EDC/NHS cross-linking method is commonly applied to stabilize the structure through ‘zero-length’ cross-links, releasing only urea derivatives as a by-product [4]. Neither EDC nor NHS is incorporated in the final material, making this method ideal for scaffold stabilization. However, during EDC cross-linking of collagen, the free primary amine groups of lysine and the carboxylic groups of glutamate or aspartate residues are used, including within the important cell adhesion sequence GxOGER. As such, this method can be detrimental for cell binding when used at high concentrations as it reduces integrin-binding site availability on the surface of the collagen-based structures [5–8]. As cell interaction with a material drives the cellular response for successful tissue regeneration, it is crucial to know how, and to what extent, various ‘degrees’ of cross-linking influence cells to find the optimal balance between stiffness and cell behaviour.

For bone tissue engineering heterotypic cell–cell contact between both osteoblasts (OBs) and endothelial cells (ECs) are known to be essential for healthy tissue regeneration allowing simultaneous bone cell growth, neovascularization and angiogenesis [1]. The delivery of oxygen through blood vessels, in particular, plays a pivotal role in cell survival. As the consumption rate of oxygen by cells is faster than its delivery by diffusion, over distances further than approximately 200 µm from the capillary lumen, metabolically active tissue requires the distance between blood vessels and cells to be within this range [9]. Therefore, appropriate cell migration and vessel ingrowth into implantable scaffolds are critical to prevent necrosis and enable successful regeneration and tissue growth *in vivo*. *In vitro* mono-culture studies are needed to gain mechanistic understanding of the response of the different cell types involved in bone healing to various scaffold properties. They provide single-variable physico-chemical data on cell responses such as cell growth, migration and cell–material interactions. Since, ultimately, in *in vivo* environments ECs and OBs grow simultaneously on the scaffold, careful tailoring of the material’s biochemical and structural characteristics to accommodate both cell types and to establish an optimal compromise between any conflicting material properties is crucial.

This work describes a systematic study to gain insight into the cell type-specific response of human osteoblast (hOBs) and human dermal microvascular endothelial cells (HDMECs) to 3D collagen scaffolds with altered biochemistry and architectural properties. This vital information could be used to inform subsequent *in vitro* co-culture assays exploring angiogenesis for bone tissue engineering.

## Materials and methods

### Substrate fabrication

Medical grade insoluble bovine dermal collagen type I (Collagen Solution, UK) was hydrated in 50 mM acetic acid (Alfa Aesar, UK) for 72 h at 4°C. The slurry was homogenized on ice at 22 000 rpm for a total of 6 min using a Waring 800EG commercial blender (Waring, USA). Air bubbles were removed by centrifuging the suspension for 5 min at 2500 rpm (Hermle Z300, Labortechnik, Germany). 2D films with a homogeneous thickness of roughly 8 µm were cast by pipetting 100 or 200 µl of 0.5 wt.% slurry into the wells of a CytoOne^®^ 96- or 48-well plate (Starlab, UK), respectively, and air-dried for 48 h. For 3D scaffolds, 1 wt.% collagen suspension was added to a 6-well tissue culture plate (TCP) or a stainless steel base mould and lyophilized in a VirTis AdVantage bench-top freeze-dryer (Biopharma Process Systems, UK) to produce isotropic and anisotropic 3D scaffolds, respectively, using the following freeze-drying parameters; collagen suspension was cooled down from 20°C to either −15, −25 or −35°C freezing temperatures with a set cooling rate of 0.9°C/min and held for 12 h before sublimation at 0°C for 30 h under a vacuum of less than 100 mTorr.

### Cross-linking

A chemical cross-linking procedure with water-soluble carbodiimide, EDC, in combination with succinimide, NHS, was used. Both 3D scaffolds and 2D films were cross-linked using 1.15 g EDC with 0.27 g NHS per gram collagen in 95% ethanol resulting in a molar ratio of 5:2:1, EDC:NHS:COO− (on collagen), hereafter referred to as 100% of the standard conditions (SC) as it results in the highest achievable mechanical stability [5, 6, 10]. The desired degree of cross-linking in 2D films and 3D scaffolds was established by immersing them in a solution diluted from the 100% SC stock solution with 95% ethanol. The scaffolds were left to cross-link for 2 h on a shaker at room temperature. 2D non-cross-linked films and 3D scaffolds were incubated in 95% ethanol. After cross-linking, both films and scaffolds were washed extensively with distilled water (15 min×5) to remove chemical traces. Finally, films were left to dry under ambient conditions, and scaffolds underwent a second freeze-drying step using the same substrate fabrication protocol as before to obtain dry, cross-linked 3D scaffolds. Dried samples were stored in the dark for up to 2 weeks before cell seeding.

### Substrate characterization

#### Morphological analysis

Structural analysis of 3D scaffolds was performed using a Skyscan 1272 benchtop Micro-CT (Bruker, Belgium) to image 4 mm diameter cylindrical, biopsy punched samples. All scans were acquired at 75× magnification, with a step size of 0.23°, a source voltage of 25 kV, source current of 200 µA, frame average of 2 and a pixel size of 3.5 µm. The flat field was corrected and no filters were used. The 3D reconstruction of the projection data was performed using the Skyscan software NRecon. No beam hardening correction was used and the threshold of min: 0.0 to max: 0.02 was applied to all scans. Before analysis, volume of interests (VOIs) of equal size (2×2×6.3 mm) were taken from the centre of each scaffold with a total volume of 25.2 mm^3^ using Skyscan software CTAn for structural analysis.

The pore size was calculated by sphere fit to z-slices using CTAn software. The degree of anisotropy (DoA) was measured using the mean intercept length (MIL) principle. The MIL method measures the number of intersection between vectors of equal length, projected in different directions, and the X–Y plane of the reconstructed scaffold section [11]. The mean intercept length is then calculated by dividing the vector length by the number of intersections. If a scaffold is anisotropic it would exhibit a different mean intercept length in different directions. Following anisotropy tensor analysis and eigendecomposition, as carried out according to da Silva *et al.* [11], the DoA can be calculated ranging from 0, indicating an isotropic structure, to 1, indicating an anisotropic structure. The percolation diameter (*d*_c_), a scale-invariant parameter described as the size of the largest spherical object which can penetrate through an infinitely large scaffold size, was measured with penetration occurring only from the top of the construct [2, 12]. The ‘Shrink wrap’ function was used to identify the volume accessible to a virtual object of diameter *d*. The accessible z-distance, *L*, follows from the relationship defined by the percolation theory as described in Equation (1) [2]:


(1) $$L=L_{0}{\left(d-d_{c}\right)}^{\nu },$$

where ν is a percolation constant with a value of 0.88 for a 3D system and *d*_c_ the percolation diameter. Values of *d* were plotted as a function of ${-L}^{\left(1/\upsilon \right)}$ to determine the percolation diameter through extrapolation of the data to infinite scaffold sizes. All parameters were measured in triplicate.

#### Mechanical testing

Compressive stress–strain analysis was performed on various 3D scaffolds with different cross-linking conditions ranging from 0% to 100% SC cross-linking using a mechanical Hounsfield tester fitted with a 5 N load cell. Scaffolds with a diameter of 8 mm and a height of 9–10 mm were hydrated in distilled water at room temperature for 1 h. Hydrated scaffolds were placed under a load cell such that the compression direction was perpendicular to the circular plane surface of the scaffold leaving a 1 mm gap. All compression tests were performed with a crosshead speed of 5 mm/min and were carried out on six replicas for each condition. Linear regression of the initial linear region of the stress–strain curve was used to obtain the linear elastic (Young’s) modulus.

For the shrinkage analysis, the height of hydrated 3D scaffolds in triplicate was measured and cultured in an incubator at 37°C using HDMEC medium. At preselected time points (1, 6, 12, 18 and 24 days) the scaffold heights were measured again and the difference calculated.

### In vitro assessment

#### Cell sourcing and handling

Cryopreserved hOBs (cat.# 406-05f, Sigma-Aldrich, UK) at passage 2 were purchased. Cells were harvested and cryopreserved at passage 4 using dimethyl sulphoxide (DMSO). The hOBs were expanded in complete cell medium (Dulbecco’s Modified Eagle Medium (DMEM, cat.# 21885025, Life Technologies Ltd., UK), supplemented with 10% (v/v) foetal bovine serum (FBS, cat.# F9665, Sigma-Aldrich, UK), 75 μg/ml ascorbic acid (cat.# A8960, Sigma–Aldrich, UK) and 1% (v/v) penicillin–streptomycin (10 000 units/ml penicillin, 10 mg/ml streptomycin)).

Pre-screened HDMECs (cat.# C-12215, PromoCell GmbH, Germany) were harvested and cryopreserved at passage 4 using Cryo-SFM (cat.# C-29910, PromoCell GmbH, Germany), a proprietary serum-free media. The HDMECs were cultured in supplemented Endothelial Cell Basal Medium MV (cat.# C-22220, PromoCell GmbH, Germany) with 15% (v/v) FBS, 1% (v/v) penicillin–streptomycin, 10 µg/ml heparin sodium salt (cat.# H3149, Life Technologies Ltd., UK), and 2.5 ng/ml bFGF (cat.# 13256029, Life Technologies Ltd., UK). Cells were maintained in a humidified atmosphere with 5% CO_2_ in air at 37°C, and culture medium was replaced every 3 days. At 70–80% confluency, cells were trypsinized with 0.25% trypsin containing 1 mM ethylenediaminetetraacetic acid (EDTA) for passaging or for seeding onto the materials.

#### Microscopy

Fluorescent visualization and analysis of stained films were performed at the Cambridge Centre for Medical Materials using a Carl Zeiss fluorescent microscope (Carl Zeiss AG, Oberkochen, Germany). Visualization of scaffolds was conducted at Geistlich Pharma AG using a spinning disk confocal microscope (CV1000, Yokogawa, Japan) equipped with a 10× Olympus UPLSApo, 10 × 2, 0.4 NA objective and a 40× Olympus UPLSApo, 40 × 2, 0.95 NA objective. Scaffolds were cross-sectioned along the vertical axis and each cross-sectioned plane was imaged up to a depth of 600 μm. Image analysis was performed using ImageJ, an open-source image processing software [13]. Cell nuclei were detected using automatic thresholding (Otsu method) in combination with the particle analysis function [14]. The Euclidean distance map function was used to quantify migration distances with the scaffolds.

#### Cell proliferation

Proliferative behaviour of hOBs and HDMECs on 2D collagen films and 3D scaffolds with various structural and surface properties was investigated. 7.5 × 10^3^ or 1.0 × 10^5^ cells were seeded on films or to the top surface of scaffolds, respectively, then cultured for 1, 3 and 7 days. Subsequently, samples were fixed with 4% paraformaldehyde (PFA, cat.# J61899, Thermo Fisher Scientific, UK) for 2 h followed by a washing step with PBS. After fixing, samples were stored at 4°C for up to 1 week before staining. Films and scaffolds were stained with 4′,6-diamidino-2-phenylindole (DAPI) or propidium iodide (PI) nuclei staining, respectively. A 1:10.000 dilution of DAPI in distilled water was applied to the films for 10 min followed by three washes with water. For PI staining scaffolds were pre-treated with RNase (1 μg/ml) for 1 h at 37°C to ensure selective DNA staining followed by three washes with PBS. A PI:wash buffer dilution of 1:250 was added to the scaffolds and incubated for 30 min and washed thoroughly with wash buffer. Both films and scaffolds were subsequently analysed using fluorescence or spinning disk confocal microscopy, respectively, for cell nuclei counting. Three samples were analysed for each condition with three random fields of view taken for each sample, resulting in nine images for each condition.

#### Cell adhesion

Cell adhesion on 2D collagen films in the presence of Mg^2+^ (total attachment) or EDTA (non-specific binding) was examined through a calorimetric measurement of lactate dehydrogenase (LDH) activity (cat.# 11644793001, Sigma–Aldrich, UK) to deconvolute non-specific and integrin-mediated binding. The presence of a divalent cation, in this case, Mg^2+^, was used to ensure metal ion-dependent integrin-based binding alongside non-integrin-mediated binding [15]. Cell adhesion in the presence of EDTA eliminates divalent cations responsible for integrin-mediated binding by chelation and thus only allows non-specific binding [8].

Non-specific attachment to the surface in the 96-well tissue culture plate containing 2D films was blocked with 5% (w/v) BSA in PBS for 1 h followed by three washing steps with PBS. Then, 200 μl of cell suspension with a density of 1.0 × 10^5^ cells/ml in serum-free DMEM, containing either 5 mM MgCl_2_ or 5 mM EDTA, was added to each well and incubated for 1 h at room temperature to allow attachment. Loosely bound cells were removed using a wash step (three times with PBS) after which 50 μl of lysis buffer containing 2% (v/v) Triton X-100 in water was added to the samples and incubated for 2 h at room temperature. After colour development upon addition of LDH substrate (50 μl), the absorbance (*A*_490_) of quadruplicate samples was measured using a FLUOstar Optima (BMG Labtech, Germany) macro plate reader. Cation-mediated attachment was estimated by subtracting the absorbance of samples in the presence of EDTA from those in the presence of MgCl_2_.

#### Cellular metabolic activity

The metabolic activity of cells within the 3D collagen scaffolds was assessed using a non-toxic dye, PrestoBlue^®^. A resazurin-based solution containing a blue, non-fluorescent cell-permeant compound was added to the samples, which converts into the red and highly fluorescent resorufin product upon metabolism in cells [16]. The absorbance was detected using an absorbance reader.

For each experimental group, three replicas alongside three blank samples (2D films/3D scaffolds without cells) were analysed. Seeded 2D films and 3D scaffolds were maintained at 37°C including a fresh culture medium change every 3 days for 24 days. At Days 1, 6, 12, 18 and 24 the supernatant was removed and discarded then the samples were placed in fresh culture medium containing 10% PrestoBlue^®^ solution and incubated at 37°C for 2 h. Subsequently, 100 μl of each sample was transferred into a 96-well tissue culture plate and the absorbance was measured at wavelengths of 560 and 590 nm, using a FLUOstar Optima (BMG Labtech, Germany) microplate reader.

#### Cell migration

Cell invasion into 3D collagen scaffolds was quantified to evaluate the influence of the material properties on cell migratory behaviour. Scaffolds fixed according to section ‘Cell proliferation’ were placed in a disposable embedding mould (Peel-A-Way, cat.# 18985, Polysciences, USA) and thoroughly immersed in 15% (w/v) pre-warmed (60°C) gelatin solution and left to solidify on ice. The mounted scaffolds were trimmed, removing excessive gelatin around the scaffolds using a blade cutter, and fixed with 10% formalin for 3 days. Lateral cross-section slices of 200 μm in thickness from the middle of each sample were cut in triplicate using a vibrating blade microtome (V1000S, Leica Biosystems, CH). The slices were stained with PI and imaged using a spinning disk confocal microscope as for section ‘Cell proliferation’.

## Results

### Physico-chemical optimization

#### Cell proliferation

**Figure 1.**
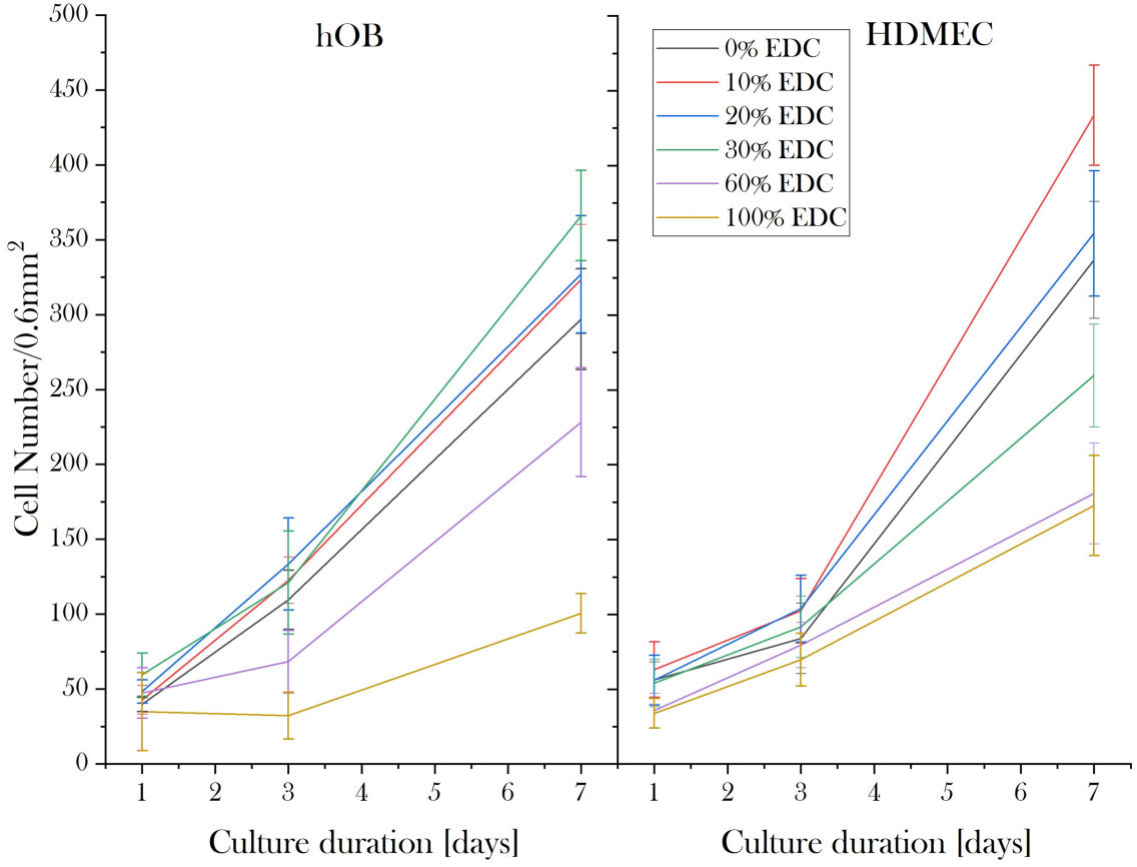
Cell proliferation profiles of hOBs and HDMECs on 2D collagen films cross-linked with increasing EDC/NHS concentrations at Days 1, 3 and 7. hOBs and HDMECs exhibited the highest proliferative capacity on 30% and 10% SC cross-linked samples, respectively. Overall, for both cell types, relatively low proliferation was detected on samples treated with 60% and 100% SC cross-linking.

The proliferative profile of both hOBs and HDMECs on 2D collagen films cross-linked with different EDC/NHS concentrations are shown in Fig. 1. A significant increase in proliferation rate with increasing cross-linking level from 0% to 30% SC was evident. Higher cross-linking levels above 30% SC reduced the proliferative capacity of hOBs, resulting in significantly lower cell numbers on these samples at Days 3 and 7 compared with those treated with lower cross-linking concentrations.

For endothelial cells, the 10% SC cross-linking treatment appeared to significantly increase cell proliferation as opposed to all other cross-linking conditions, exhibiting the highest cell number at 7 days of incubation. A steep decline in cell numbers was detected with EDC/NHS cross-linking concentrations above 10% SC cross-linking. HDMECs seeded on 2D films cross-linked with 60% and 100% SC cross-linking, in particular, exhibited a low proliferative capacity.

#### Cell adhesion

**Figure 2.**
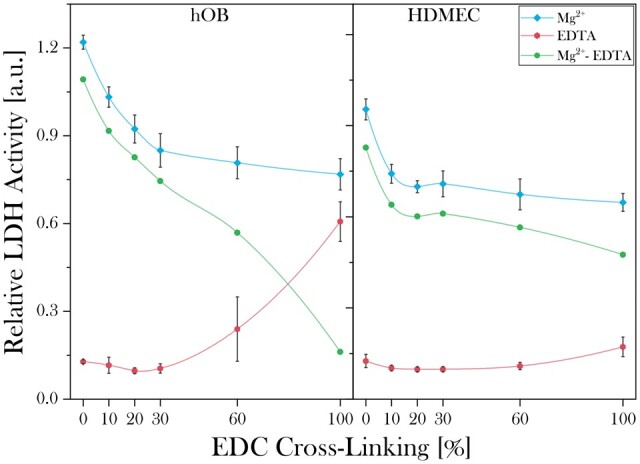
Mg^2+^-dependent, non-specific (EDTA) and integrin-mediated (Mg^2+^–EDTA) adhesion profiles of hOBs and HDMECs on 2D collagen films with different cross-linking levels. For both cell types, specific binding decreases with increasing cross-linking levels with hOBs showing a higher sensitivity than HDMECs to cross-linking levels above 30% SC. An increase in non-specific binding could be seen for hOBs at higher cross-linking levels, while this binding mechanism was insignificant for HDMECs.

Cell adhesion profiles in the presence of Mg^2+^ (total), EDTA (non-specific) and the consequent integrin-specific (Mg^2+^–EDTA) attachment on 2D collagen films treated with different degrees of cross-linking are shown in Fig. 2. For osteoblasts, total binding in the presence of Mg^2+^ was the highest for 2D non-cross-linked films. The native binding on collagen was reduced by around one-third when increasing from 0% to 30% SC cross-linking. Only 14% of the binding activity was retained on 100% SC cross-linked 2D films over a non-cross-linking control. Surprisingly, the non-specific attachment detected in the presence of EDTA increased significantly at cross-linking levels above 30% SC.

Similar to hOB attachment, HDMECs exhibited the highest total cell attachment on non-cross-linked 2D films which reduced significantly with increasing cross-linking levels, reaching a plateau at 20% SC. No non-specific binding was apparent between 0% and 60% SC cross-linking and only a minor increase was detected for 100% SC cross-linked 2D films. Comparatively, initial hOB attachment shows a higher sensitivity than HDMECs to cross-linking levels above 30% SC.

#### Metabolic activity

**Figure 3.**
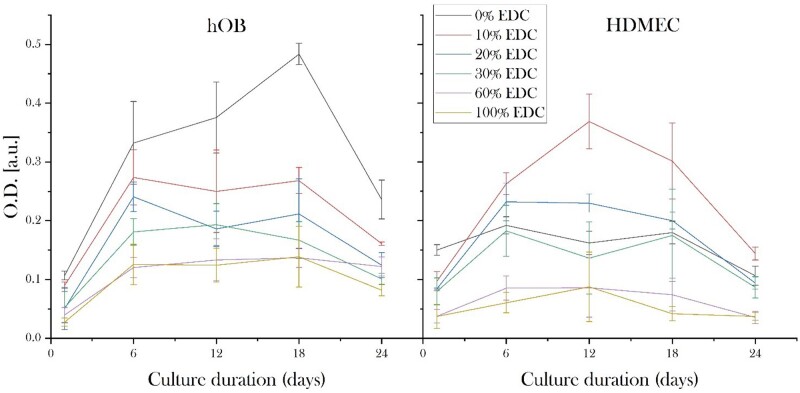
Plot representing metabolic activity of hOBs and HDMECs seeded on isotropic 3D collagen scaffolds with various cross-linking degrees at Days 1, 6, 12, 18 and 24. A negative correlation between cross-linking levels above 10% SC and metabolic activity could be seen for both HDMECs and hOBs. hOBs and HDMECs exhibited the highest activity on 0% and 10% SC cross-linked samples, respectively.

The metabolic activity of hOBs and HDMECs mono-cultured on isotropic 3D collagen scaffolds of various cross-linking levels over 24 days in culture are shown in Fig. 3. For osteoblasts, at all time points, the highest metabolic activity was found on non-cross-linked scaffolds, which reduced dose-dependently with increasing cross-linking. The first 6 days were found to result in the most significant increase in cellular activity for all samples with the highest activity achieved between Days 6 and 18 for all hOB seeded samples. The metabolic activity of hOBs on 60% and 100% SC cross-linked scaffolds was lower at all time points compared to 0–30% cross-linked scaffolds.

For HDMECs, at Days 16–18, 10% SC cross-linked samples supported the highest metabolic activity followed by an EDC-dependent decrease in activity at higher cross-linking levels. Notably, both 10% and 20% SC cross-linked scaffolds induced enhanced activity compared with non-cross-linked samples. For higher cross-linking levels of 60% and 100% SC, however, the metabolic activity was reduced compared to all other conditions and remained low for all time points. Within each cross-linking condition, the metabolic activity peaked at either Day 6 or 12. A comparison between the two cell types revealed that both HDMECs and hOBs had high metabolic activity on 20% and 30% SC cross-linked scaffolds.

#### Scaffold shrinkage

**Figure 4.**
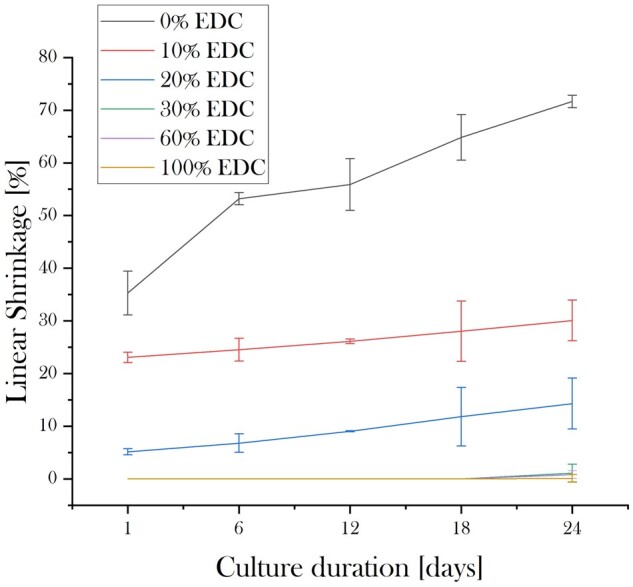
Linear z-dimension shrinkage of isotropic 3D collagen scaffolds, treated with different EDC/NHS concentrations, after Days 1, 6, 12, 18 and 24 of incubation in culture medium. Non-cross-linked, 10% and 20% SC cross-linked scaffolds showed significant shrinkage over the incubation period, with the majority of the shrinkage occurring within the first 24 h. Scaffolds with a 30% SC cross-linking or higher showed marginal shrinkage after long term incubation of 24 days.

The shrinkage of isotropic 3D collagen scaffolds with different cross-linking levels under regular culture conditions is displayed in Fig. 4. Non-cross-linked scaffolds shrank significantly over the total period of the experiment by approximately 70% of their original height, where half of the shrinkage occurred within the first 24 h. The majority of shrinkage of 10% SC cross-linked grafts also took place within 24 h but was markedly lower than for the non-cross-linked samples. Scaffolds with 30% SC cross-linking or higher showed little shrinkage which occurred only after a long term incubation of 24 Days.

#### Cell migration

**Figure 5.**
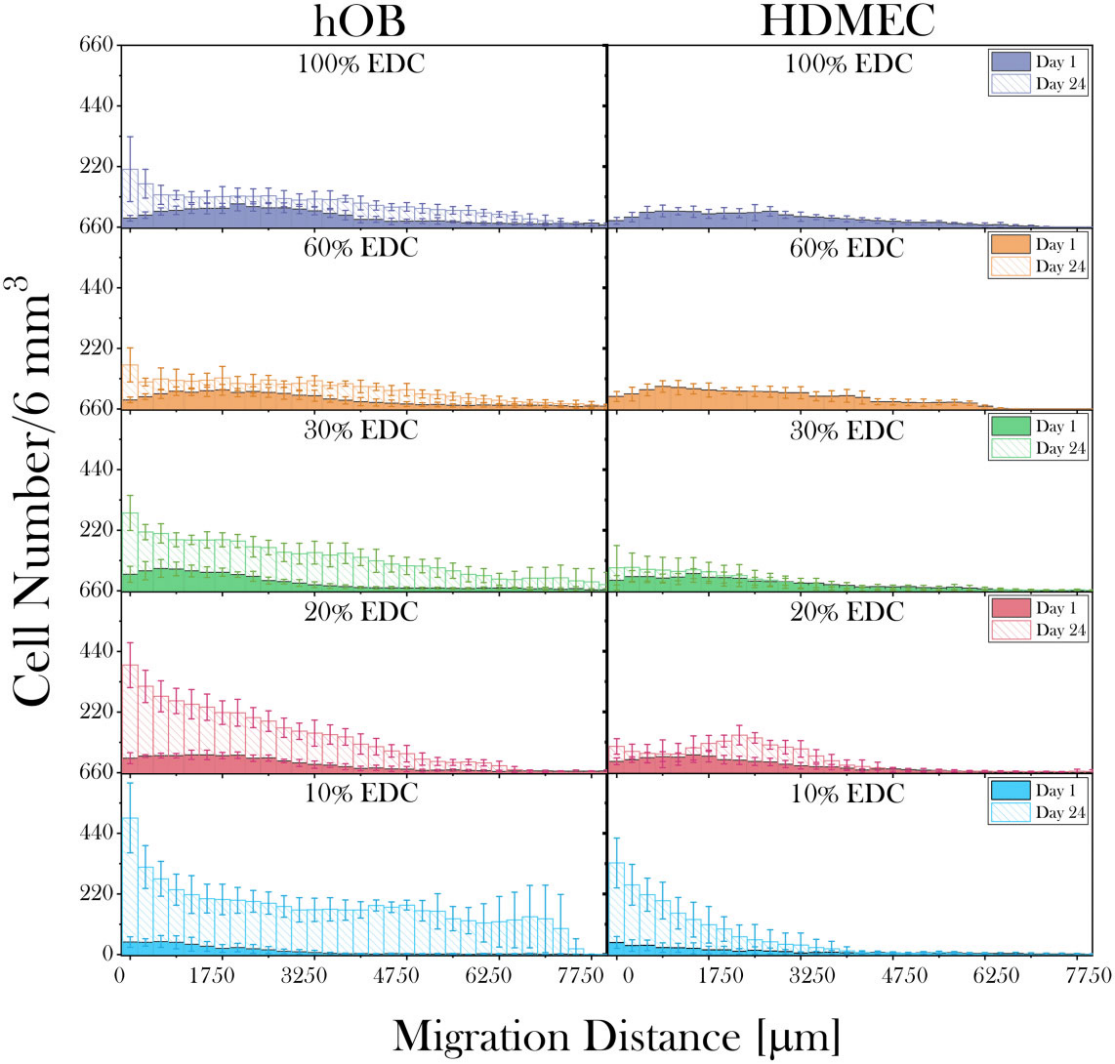
Graph illustrating the migration trend of hOBs and HDMECs seeded on EDC/NHS treated isotropic 3D collagen scaffolds between Days 1 and 24. For both cell types, higher cell numbers were detected on lower cross-linking levels. Overall, hOBs exhibited an elevated cell invasion and proliferation capacity compared with HDMECs.

The invasion profiles of primary hOBs and endothelial cells into isotropic 3D collagen scaffolds treated with various EDC concentrations over a period of 24 days are shown in Fig. 5. This shows that the cross-linking degree impacts the migration profiles for both cell types. While on Day 1 the infiltration of hOBs increased with increasing cross-linking levels, migration was observed to be higher at lower cross-linking levels as detected by the migration distance measured at Day 24. Moreover, on Day 24, reduced cell numbers were detected on scaffolds with increasing cross-linking levels. Within each condition at prolonged incubation, the highest cell number was obtained closest to the scaffold surface, with 10% SC EDC treated scaffolds maintaining a relatively high cell number over the entire scaffold thickness.

While the hOBs were found to be evenly distributed over the measured total distance for 10% SC cross-linked samples, the majority of HDMECs on these samples travelled only around one-fifth of the total distance. On 20% SC cross-linked samples, HDMECs migrated furthest with a cluster of cells close to the centre of the scaffold on Day 24. No HDMECs were detected in both 60% and 100% SC cross-linked samples at Day 24 indicating cell death. In general, hOBs demonstrated elevated cell invasion and proliferation compared with HDMECs.

#### Mechanical testing

**Figure 6.**
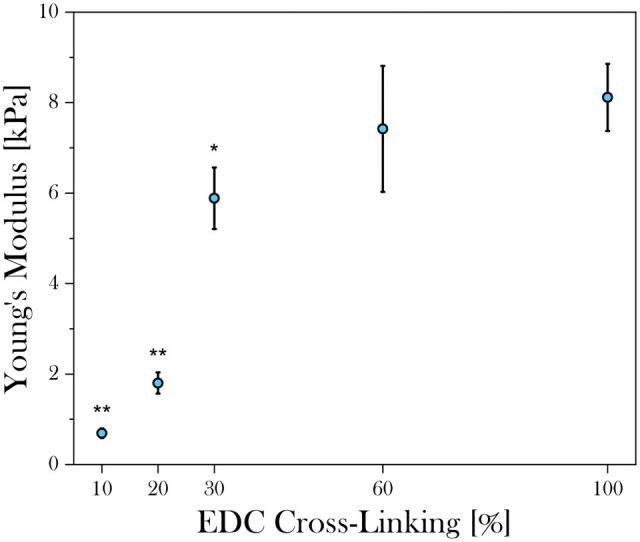
Young’s modulus of the low-strain linearly elastic region of hydrated isotropic 3D collagen scaffolds with cross-linking levels ranging between 10% and 100% SC. An increase in cross-linking degree from 20% to 30% SC had the greatest impact on the Young’s modulus resulting in an increase of about 4 kPa. About 60% and 100% SC increased the Young’s modulus to ∼7 and 8 kPa, respectively, showing a reduced influence of cross-linking treatment on the mechanical stiffness at higher levels. A double asterisk indicates a significant difference with 30%, 60% and 100% SC (*P* < 0.01). a single asterisk indicates a significant difference with 100% SC (*P* < 0.05).

As seen in Fig. 6, compressive stress–strain analysis of hydrated 3D collagen scaffolds with different degree of cross-linking revealed a relatively low Young’s modulus of approximately 1 kPa for 10% SC and 2 kPa for 20% SC cross-linked scaffolds. In a non-linear relationship with EDC concentration, there was a steep 3-fold increase to 6 kPa with a 20% increase in SC cross-linking. A further rise in cross-linking degree to 60% and 100% SC cross-linking did not significantly increase the mechanical stiffness any further resulting in Young’s moduli of about 7 and 8 kPa, respectively.

### Biological response to architectural features

#### Morphological analysis

A systematic assessment of the hOB and HDMEC response to structural features was carried out. Six 3D scaffolds with different structural properties were obtained using either a 6-well tissue culture plate or a stainless steel base mould with altering freezing conditions as summarized in Table 1, with S_2_ being comparable to the scaffolds used in the previous sections. A 30% SC cross-linking treatment was applied to all scaffolds to achieve an appropriate balance between mechanical stability and cellular response. As seen in Table 1, the moulds allowed for the production of two distinct types of constructs exhibiting lower DoA values with larger pores and *d*_c_*s* (S_1−3_) or slightly higher DoA values displaying significantly smaller pores and percolation diameters (S_4−6_). For the 6-well plate fabricated samples the pore size decreased with decreasing temperature, however, for the steel-base mould fabricated samples the freezing temperature did not influence the pore size.

**Table 1.** Architectural parameters of 3D scaffolds freeze-dried using different moulds and freezing temperatures

Name	Mould	Freezing T. (°C)	Pore size (μm)	*d*_c_ (μm)	DoA
S_1_	6-well plate	−15	175 ± 2.5	130 ± 26.7	0.38 ± 0.08
S_2_	6-well plate	−25	150 ± 12.2	3147 ± 12.4	0.46 ± 0.06
S3	6-well plate	−35	140 ± 4.8	120 ± 9.8	0.55 ± 0.08
S4	Steel base	−15	81 ± 3.0	94 ± 6.0	0.57 ± 0.03
S_5_	Steel base	−25	82 ± 7.3	88 ± 9.0	0.67 ± 0.04
S_6_	Steel base	−35	80 ± 7.6	76 ± 6.9	0.62 ± 0.03

The protocols resulted in the production of two distinct groups of constructs exhibiting lower DoA values with larger pores and *d*_c_*s* (S_1 − 3_) or slightly higher DoA values displaying significantly smaller pores and percolation diameters (S_4 − 6_).

Representative 2D cross-sections and top views are depicted in Table 2. S_1−3_, exhibited clear isotropic features and the pore sizes were relatively large as compared with S_4−6_ which exhibited narrow directional pore structures. Pore homogeneity was observed for the isotropic 3D scaffolds S_2_ and S_3_. For the anisotropic 3D scaffold S_5_, the pore size distribution was found to be relatively homogeneous. S_1_, S_4_ and S_6_ exhibited a clear heterogeneous pore size distribution with all having significantly smaller pores at the bottom of the scaffold as opposed to the centre and top segment. Important to note is that despite the similarities in the mean pore values as previously mentioned for S_4−6_, significant variation in the overall pore structure was distinguished. This can be partly attributed to the slight pore heterogeneity and partly to the differences in alignment. S_4_ appeared to exhibit relatively large pores at the centre of the scaffold with a minor cluster of small pores at the bottom and alignment restricted to the top segment of the scaffold. In contrast, S_5_ possessed small pores with high directionality throughout the entire structure. For S_6_, besides having the most significant pore heterogeneity, pore alignment was restricted to a small segment at the top of the scaffold as compared with S_4_.

**Table 2.** Top views and cross-sections of the VOIs (2×2×6.3 mm) of the 3D collagen scaffold structures produced with different moulds and freezing temperatures. S_1−3_ revealed clear isotropic features with larger pores as opposed to the aligned scaffolds S_4−6_ exhibiting narrow pore structures. The highest pore homogeneity could be seen for S_2_ and S_3_, followed by S_5_. The most apparent pore size heterogeneity could be observed for S_1_, S_4_ and S_6_.

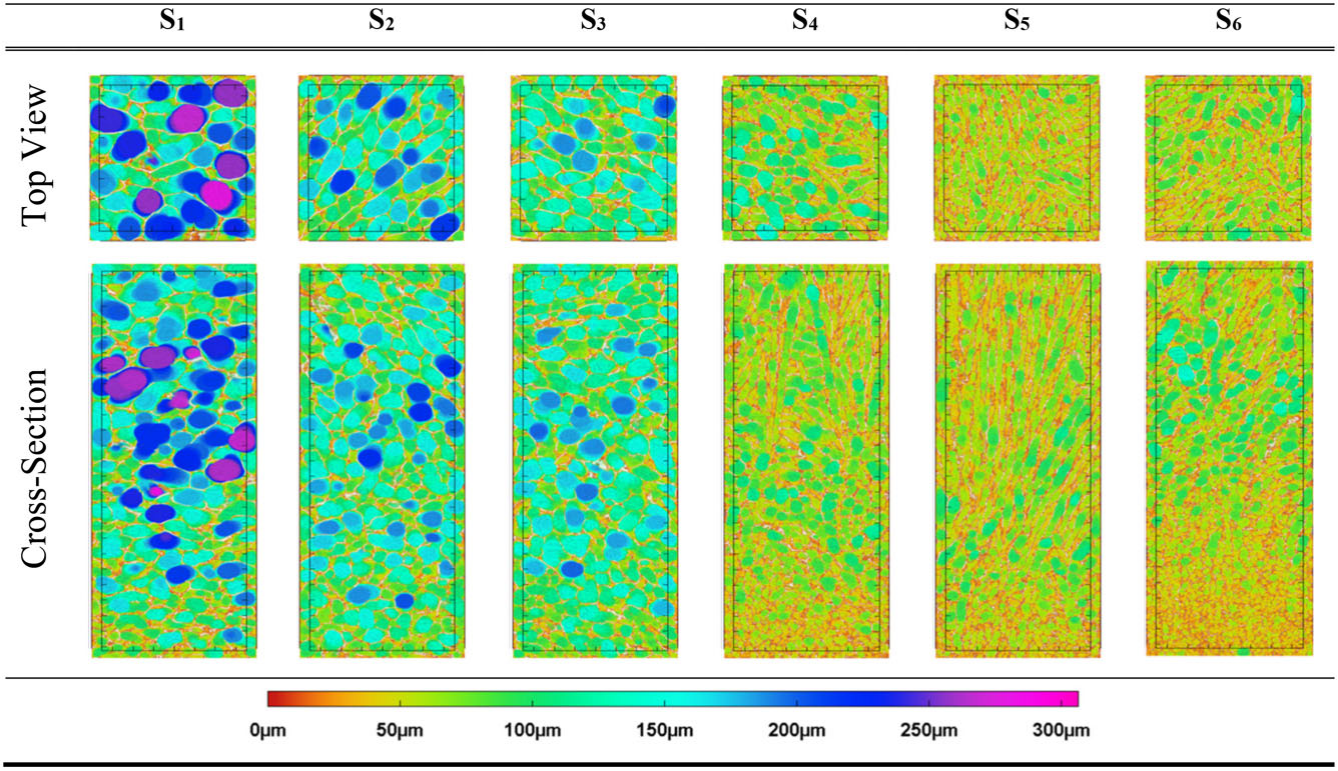

#### Migration behaviour of hOBs in response to structural features

**Figure 7.**
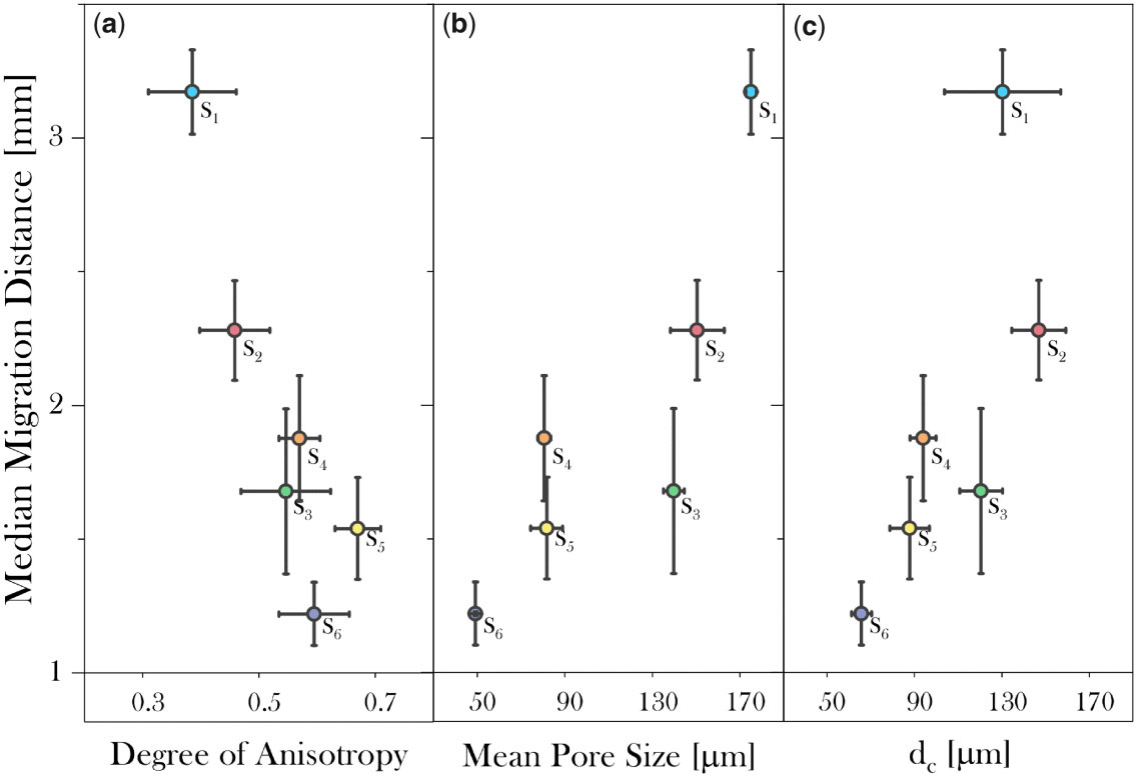
Plot representing the median migration distance of hOBs at Day 18 on 30% SC cross-linked collagen scaffolds with different architectural features; DoA (a), mean pore size (b) and percolation diameter (c). for S_1 − 3_, general patterns can be extracted showing a decrease in median migration distance with increasing scaffold alignment and median pore size. For S_4 − 6_, no well-defined correlation could be extracted between the migration distance and the DoA, but the migration appeared to increase with increasing *d*_c_.

Correlational analysis between median distance travelled by osteoblasts and the DoA (a), the mean pore size (b) and the percolation diameter (c) was carried out on various 3D scaffolds as seen in Fig. 7. As hOBs were found to be most metabolically active at Day 18 on 30% SC cross-linked scaffolds, this was taken as a suitable time point to assess the cellular response. From the DoA data a general pattern showing a decrease in median migration distance with increasing scaffold alignment could be seen, particularly for the isotropic moulds S_1−3_. This correlation became much less apparent for scaffolds with higher DoA values, S_4−6_. Nevertheless, from the data obtained, it is not possible to assign an independent influence of DoA on cell migration, as an increase in DoA is associated with an overall decrease in mean pore size and *d*_c_. To illustrate this co-dependency of architectural features, hOBs migrated further into S_1_ which has the lowest DoA but also the largest mean pore size and one of the largest percolation diameters. Conversely, the lowest migration distance was obtained for S_6_ which exhibited nearly the highest DoA but had the lowest mean pore size and *d*_c_.

Production of the three aligned scaffolds successfully eliminated mean pore size as a variable for S_4_ and S_5_, as these showed the same pore size (Table 1) allowing delineation of the impact of *d*_c_ and DoA on the migration distance of hOBs. Although, for the aligned scaffolds, no well-defined correlation could be extracted between the migration distance and the DoA, the migration was found to increase steadily with increasing *d*_c_. Interestingly, the hOBs seeded on S_3_ were limited in their migration despite having a similar DoA value, larger mean pore size and *d*_c_ compared with S_4_. However, even though the average DoA value is similar between these scaffolds, the pores at the top of S_3_ were significantly more isotropic than for S_4_, as seen in Table 2, indicating the importance of pore alignment for cell migration.

**Figure 8.**
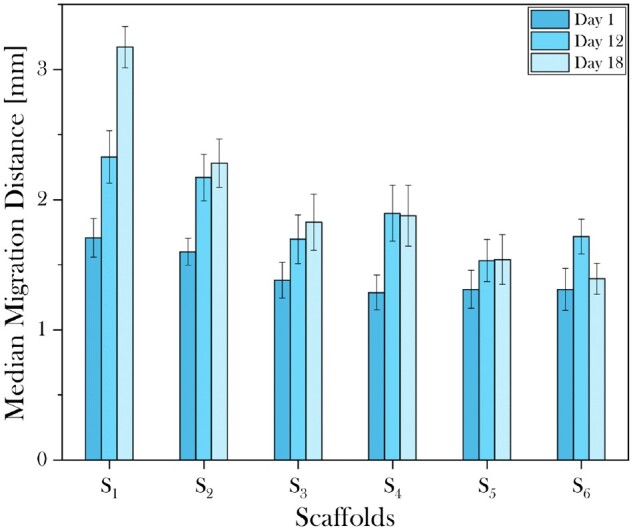
Plot representing the median migration distance of hOBs cultured on 30% SC cross-linked collagen scaffolds with different architectural features at Days 1, 12 and 18. Particularly for isotropic scaffolds S_1 − 3_, the increase in incubation time positively influenced the migration behaviour of hOBs. Anisotropic scaffolds S_4 − 6_, induced the same behaviour over a time span of 12 days, but longer incubation did not result in increased migration.

The hOB cell infiltration in all scaffolds over 18 days in culture revealed that, in general, a longer incubation time was associated with increased migration, especially in isotropic samples which possessed larger pores and percolation diameters (Fig. 8). Overall, the infiltration distance of hOBs into the scaffolds at Day 1 appeared to be similar between all anisotropic scaffolds (S_4−6_) and was found to increase slightly when comparing S_1_ to S_3_. The most significant increase in the migration distance over time was measured for S_1_, having the largest mean pore size and one of the highest *d*_c_ values, indicating active migration through this scaffold. This impact of incubation time on migration distance progressively declined with decreasing mean pore size and *d*_c_ as seen for S_2_ and S_3_. Anisotropic scaffolds S_4−6_, all possessing similar mean pore values, showed inconsistent trends regarding migration distance over time. Despite exhibiting a decrease in *d*_c_ moving from S_5_ to S_6_, at Day 12, increased migration was observed on S_6_ as opposed to S_5_. The subsequent marked decrease in migration distance observed for S_6_ at Day 18, resulted in the positive correlation between *d*_c_ and hOB migration in Fig. 7. For both S_4_ and S_5_ longer incubation times above 12 days did not result in additional cell migration.

#### Invasion behaviour of hOBs and HDMECs

**Figure 9.**
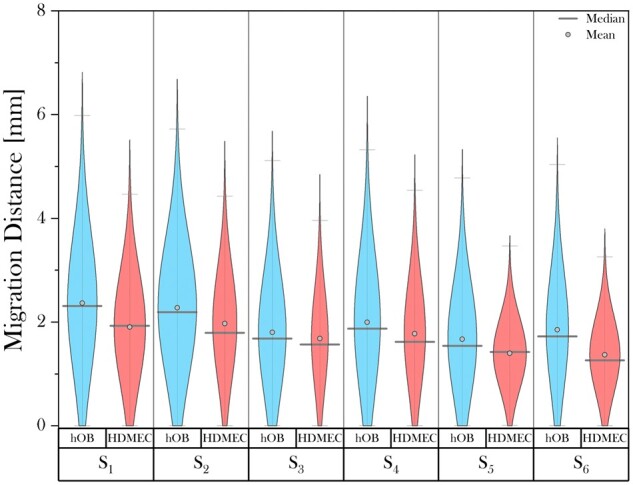
Graph representing the migration distribution of hOBs and HDMECs into 30% SC cross-linked collagen scaffolds at 12 days of incubation. Within all scaffolds, osteoblasts travelled further than endothelial cells in terms of median and furthest cell distance travelled. Within both isotropic and anisotropic scaffolds, the median distance travelled for hOBs and HDMECs decreased gradually with declining mean pore size for S_1 − 3_ and decreasing *d*_c_ for S_4 − 6_.

Cell type-specific migration was examined for both hOBs and HDMECs, seeded on the previously analysed 3D scaffolds. The cell invasion assay was performed on Day 12, as metabolic activity analysis indicated high activity for both osteoblasts and endothelial cells at this incubation time. Figure 9 illustrates the normal distribution of both hOBs and HDMECs migrating into scaffolds S_1−6_. Within all scaffolds, osteoblasts migrated further than the endothelial cells in terms of median and furthest cell travelled. Similarly to hOBs, within both isotropic and anisotropic scaffolds, the median distance travelled by HDMECs decreased gradually with decreasing mean pore size for S_1−3_ and decreasing *d*_c_ for S_4−6_. The mean pore size or the percolation diameter had a greater impact on hOBs than HDMECs. Interestingly, the relatively low distance travelled as well as the distinct cell distribution obtained for HDMECs invading S_5_ and S_6_ indicated accumulation of cells at a shorter distance as opposed to all other scaffolds.

## Discussion

The architecture, surface biochemistry and stability of 3D scaffolds provide important cues that modulate cell behaviour. This study aimed to optimize scaffold properties to support hOB and HDMEC cells independently in a 2D and 3D environment. The aim was to establish a general understanding of the influence of the scaffold properties on the cellular response as shown schematically in Supplementary Fig. S1.

### 2D analysis

#### Cell adhesion and proliferation

Stabilization of collagen grafts with EDC/NHS has expanded their application, as it allows scaffolds to endure structural manipulation before transplantation, resist cell contraction during healing, withstand external pressure after implantation and prevent scaffolds from collapsing [17–19]. However, as this method has been found to reduce cellular activity and cell viability, a compromise between mechanical properties and biological functionality is required [5, 8]. Cells naturally bind to collagen via integrins α_1_β_1_, α_2_β_1_, α_10_β_1_ and α_11_β_1_, interacting with ligands through the I-domain located in the α subunit [20]. The metal-ion-dependent adhesion site (MIDAS) located in the I-domain coordinates collagen binding through bound divalent cations such as Mg^2+^ [20, 21]. The addition of the divalent cation chelator, EDTA, removes the Mg^2+^ ion from the MIDAS site in the I-domain, disrupting native-like metal-ion-dependent and allowing only non-physiological metal-ion-independent cell binding to cross-linked collagen.

Consistent with the literature, the results obtained showed a decrease in metal-ion-dependent binding with increasing cross-linking level for both cell types. This could be attributed to the loss of free carboxylic acid groups that are essential for integrin-mediated-cell binding that are located on the ‘E’ of the consensus GxOGER cell binding motif within collagen [5]. Interestingly, this EDC dose-dependent decrease in cell binding observed after 1 h of incubation was not reflected in the cell proliferation data after prolonged incubation. For both cell types, 30% and 10% SC cross-linking levels were found to increase the proliferation rate of hOBs and HDMECs, respectively, while higher cross-linking levels inhibited proliferation. As widely recognized, substrate stiffness plays a key role in tailoring cell behaviour, with osteoblasts reportedly exhibiting a more mature phenotype and improved attachment and proliferation on stiffer surfaces as opposed to endothelial cells which function and maintain their phenotype on softer substrates [22–24]. It can be postulated that prolonged incubation allows cells to effectively sense surface stiffness through the development of actin stress fibres, which becomes the dominant factor influencing cell behaviour, thereby partially counteracting reduction in integrin-mediated cell binding sites with increasing EDC/NHS cross-linking.

Furthermore, a high rise in metal-ion-independent binding was recorded for hOBs and a minor increase for HDMECs as the cross-linking level increased. This was consistent with Bax *et al.* [5] who reported a similar trend for Rugli, HT1080 and integrin-transfected C2C12 cells. To date, it remains unclear why non-specific binding of hOBs and HDMECs increase with increasing cross-linking levels. Nonetheless, the preferable native-like binding was predominant on 2D films cross-linked up to 30% SC for both hOBs and HDMECs, indicating physiologically relevant cell binding. Therefore, a maximum cross-linking level of 30% SC is believed to result in appropriate cell adhesion and proliferation for both hOBs and HDMECs.

### 3D analysis

#### Degree of anisotropy

The DoA for all isotropic samples (S_1−3_) produced using a 6-well plate revealed a relatively equiaxed structure. A uniform mould is believed to induce the same ice crystals growth rate in all directions causing the formation of isotropic pore structures within the scaffolds produced, as described by Davidenko *et al.* [25]. For anisotropic scaffolds S_4−6_, S_4_ exhibited a surprisingly isotropic DoA value despite the variable thermal conductivity between the base and the walls which are made of different materials. This result contradicts previous findings through SEM analysis obtained by Davidenko *et al.* [25] using the same mould design. The conductive base and insulating walls of moulds producing samples S_4−6_ is expected to have induced a vertical thermal gradient upon freezing resulting in variable crystal growth in different directions and, therefore, induced the formation of anisotropic structures, which was also confirmed by Davidenko *et al.* [25]. Only S_5_ appeared to result in anisotropic structures. Nevertheless, it is important to note that the DoA describes the average alignment within a scaffold. After visual inspection of the internal structures, an isotropic structure was observed at the bottom of S_4_ and S_6_ while strong alignment was observed at the top resulting in an overall isotropic DoA value.

#### Mechanical stiffness

Mechanical testing revealed that EDC/NHS cross-linking increased the stiffness of 3D collagen scaffolds. The most striking increase in scaffold stiffness was obtained when increasing from 10% to 30% SC cross-linking. The subsequent increase in stiffness decreased with increasing cross-linking levels, most likely due to saturation of the cross-linking reaction.

Although all collagen scaffolds exhibited Young’s moduli which were orders of magnitude lower than the natural stiffness of bone (≈1 GPa), scaffolds with stiffnesses lower than the 6 kPa obtained for the 30% SC cross-linked scaffolds assessed here, have been previously found to induce appropriate osteoblast proliferation, having a stiffness of 1.25 kPa, while even more compliant substrates with stiffness of about 0.45 kPa were observed to enhance the maturity of osteoblasts to a mineralizing phenotype [26]. Navarrete *et al.* [23] also recognized that for appropriate osteoblast attachment the conventional dogma ‘the harder, the better’ is not necessarily true, reporting significantly impaired hOB behaviour on substrates with a Young’s modulus closer to that of the cortical bone. A possible explanation for these observations might be the naturally dynamic ECM stiffness for OBs during bone formation. Initially bone is formed as a softer, pre-mineralized collagenous matrix (30–100 kPa) followed by ECM stiffening over time as a result of OB matrix secretion and mineralization [27, 28]. Therefore, OB maturation often occurs in a relatively soft matrix that is more similar to the stiffness of 3D collagen scaffolds. To date, no consensus exists about the optimal stiffness of scaffolds for osteoblasts and, as cell behaviour strongly depends on substrate type, cell-ligating sequences, structure and various other conditions, focusing solely on scaffold stiffness may not be useful [23, 26, 27].

The shrinkage data showed a significant reduction in the scaffold’s height within the first 24 h, and more substantially after 24 days, in the absence of cross-linking agents, highlighting the importance and necessity of stabilization via cross-linking. The degree of shrinkage reduced with an increasing concentration of cross-linker where 30% SC cross-linking was found to be sufficient for producing scaffolds that did not shrink over 28 days in culture.

#### Cell proliferation and metabolic activity

The proliferation and metabolic activity trends of HDMECs seeded on 3D collagen scaffolds cross-linked to different degrees was in accordance with the trends observed in 2D, showing maximal proliferation with 10% SC cross-linking. In contrast, while in 2D the proliferation of hOBs improved with increasing cross-linking levels up to 30% SC, in 3D both their metabolic activity and proliferative capacity were found to progressively decline with increasing scaffold cross-linking. Although non-cross-linked scaffolds resulted in appropriate metabolic activity for both cell types, significant shrinkage occurred within the first 24 h, resulting in a loss of more than 90% of the original volume over the course of the experiment. Conversely, significantly improved structural stability induced by EDC/NHS cross-linking prevented scaffold shrinkage, but with correspondingly lower cellular activity. Nevertheless, hOBs and HDMECs seeded on scaffolds with cross-linking levels up to 30% SC exhibited appropriate metabolic activity. The highest increase in metabolic activity was observed during the first 6 days of incubation for all samples. The reduction in metabolic activity for HDMECs after Day 12 may be explained by the inability of endothelial cells to maintain cellular viability and proliferative capacity during long term mono-cultures, as previously reported [29]. For hOBs, despite relatively high cell numbers, measured by microscopy-based proliferation analysis on 0%, 10% and 30% SC cross-linked scaffolds at Day 24, a surprisingly low cellular metabolic activity was observed after 24 days of incubation. This discrepancy may be attributed to downregulated metabolism of cells after reaching confluency resulting from contact inhibition of cell proliferation [30].

#### Cell migration

For HDMECs, although the highest overall cell number was obtained on 10% SC cross-linked 3D scaffolds the majority of the cells were clustered together at the top 2 mm region of the scaffold. Generally, endothelial cells formed clusters on both 10% and 20% SC cross-linked scaffolds. This behaviour may be attributed to the particular importance of intercellular junctions for cellular communication and cell survival for HDMECs [31]. Overall, HDMECs showed limited cell migration. In future, use of co-culture experiments incorporating osteoblasts may support endothelial cell migration as endothelial cells require support from other cell types to successfully migrate and organize into microcapillary-like structures [29, 32, 33]. The observed cell death for HDMECs after 24 days of incubation on 60% and 100% SC cross-linked scaffolds may be attributed to the loss of cell binding sites on collagen during cross-linking in combination with a potentially unfavourable stiffness as was observed in a 2D environment. For hOBs and HDMECs, increased infiltration was obtained with increasing cross-linking levels at Day 1. This could be associated with the structural rigidity of the scaffolds improving their stability under wet conditions thereby enabling cells to penetrate deeper into the construct upon seeding, which is most likely a result of the capillary forces rather than active cell migration. By contrast walls of lower cross-linked scaffolds tend to collapse, limiting the capillary forces and pore accessibility for seeded cells. For both cell types, cross-linking levels higher than 30% SC significantly reduced cell migration and proliferation. Therefore, despite the improved scaffold rigidity, the loss of binding sites inevitably affected cell behaviour in the long term.

Taken together, scaffolds cross-linked between 10% and 30% SC supported both hOBs and HDMECs while providing appropriate stability to prevent the scaffolds from collapsing. Nevertheless, 30% SC cross-linking resulted in significantly improved mechanical strength as opposed to 10% and 20% SC, making this the most suitable cross-linking condition for bone tissue engineering.

### Biological response to architectural features

Interrelated scaffold architectural parameters, such as pore size, interconnectivity and pore alignment provide the spatio-temporal conditions that control cell migration, remodelling and ultimately stimulating appropriate integration with native tissue, which is essential for the success of tissue-engineered grafts. Various studies have examined the relationship between specific structural features and cell behaviour, but as the different structural parameters are not truly independent of one another, it has proven difficult to draw firm conclusions about the precise effect of each parameter [3, 34, 35]. This study attempts to consider the combined effect of all structural parameters to better understand the cell type-specific response to architectural features.

#### Cell migration in response to architectural features

Scaffolds were fabricated exhibiting different pore directionality, pore sizes and percolation diameters. These showed that median hOB migration over 18 days declined with increasing structural alignment and decreasing mean pore values and *d*_c_. By far the longest migration distance was obtained for the most isotropic scaffolds with the largest mean pore size and one of the highest percolation diameters. The importance of large pores has been observed previously for ceramic scaffolds where osteogenesis increased in scaffolds with pore sizes larger than 300 μm [36]. At Day 18, the similar *d*_c_ values observed for S_1_ and S_2_ did not seem to disrupt the practically linear correlation observed between cell invasion efficiency and mean pore size/DoA, showing an increase in migration distance of almost 1 mm moving from S_2_ to S_1_. At Days 1 and 12, however, the *d*_c_ observed for S_1_, comparable with S_2_, did appear to impede migration, with S_1_ and S_2_ exhibiting roughly similar migration distances. Since the pore homogeneity was comparable for all three scaffolds, these findings may indicate that while at first *d*_c_ is the determining factor for cell migration, over time mean pore size becomes a more critical feature. This trend could be related to the fact that cells aggregate over time potentially occluding the porous structure, thereby restricting accessibility of pores for both cells and nutrients in a pore size dependent manner, as suggested by Murphy *et al.* [3]. As all pore sizes initially possessed sufficient free volume for cells and nutrient exchange, the similar *d*_c_ observed for S_1_ and S_2_ was the limiting factor inducing comparable migration behaviour. However, after Day 12 of incubation, cell aggregation may have begun to restrict further cell invasion for S_2_, as this exhibited a smaller pore size, which required less occlusion than the other scaffolds, thereby preventing migration/nutrient exchange. By contrast cell aggregation may not restrict the porosity of the larger-pored S_1_ to a sufficient extent to restrict cell migration. These findings suggest that the impact of architecture on migration distance is a result of a complex combination of factors that may exhibit a temporal effect on cell behaviour. Reliable assessment of the influence of architectural features on cell behaviour, therefore requires a temporal assessment of cell behaviour.

Anisotropic scaffolds were fabricated that exhibited similar mean pore sizes, but different percolation diameters and cells were seeded parallel to the alignment. This increasing percolation diameter, independent of pore size, seemed to impact cell migration, showing increased migration with increasing *d*_c_. The importance of maximizing the *d*_c_ in aligned collagen scaffolds has been reported previously showing that a *d*_c_ of at least 40 μm is necessary to induce efficient cell migration [37]. However, the cell migration trend in response to percolation diameter observed here was non-linear which may be explained by additional factors such as the difference in pore homogeneity and DoA. This is evident at the top segment of S_4_ which possessed bigger and more isotropic pores, and S_5_ which exhibited much smaller and more anisotropic pores compared with S_6_. The top section is effectively the pore structure initially sensed by the surface-seeded cells. Therefore, the attributes of this top section most likely positively influenced the cell invasion observed in S_4_ and/or restricted the cell migration to some degree in S_5_. In fact, the lowest migration was initially observed for S_5_ instead of S_6_ at Day 12 which may be explained by the discrepancy in pore homogeneity. At later stages, the internal structure of S_6_, exhibiting highly irregular clusters of small and large pores at various regions, combined with a lower percolation diameter, may have restricted cell migration as well as cell survival over time, eventually resulting in a lower mean migration distance than S_4_ and S_5_. In general, all observed trends were found to result from a combination of factors exhibiting a temporal impact on cell migration behaviour.

Comparing between isotropic and anisotropic scaffolds, it was interesting to observe that the relatively high mean pore size and percolation diameter for isotropic scaffold S_3_ resulted in a lower migration distance compared to the anisotropic scaffold S_4_. Due to the distinct internal structure of the isotropic scaffolds S_1−3_, as opposed to the anisotropic scaffolds S_4−6_, this trend highlights that the pore size, percolation diameter and DoA alter cell migration differently between anisotropic and isotropic structures. This is supported by the distinct trends observed between the two scaffold groups (TCP and metal base fabrication mould) with increasing pore size and *d*_c_ appearing to have a stronger impact on aligned structures as opposed to non-aligned samples. A previous study carried out by Ashworth *et al.* showed that periodontal ligament fibroblast cell migration did not improve significantly in anisotropic scaffolds with a pore size larger than 100 μm when the percolation diameter was optimized, while isotropic scaffolds generally require pore sizes over 75–100 μm [36, 37]. Additionally, in anisotropic scaffolds cells appeared to respond more strongly to differences in pore alignment than to the changes in pore size [37]. Overall, pore alignment appeared to result in elevated migration despite having significantly smaller pores. As both pore alignment, with cells seeded parallel to the alignment, and pore size positively influence cell migration, future studies should examine aligned structures with bigger pore sizes as this may result in more extensive migration in comparison to the currently obtained isotropic structures due to the straighter route for cells on aligned scaffolds.

#### Cell invasion efficiency comparison between hOBs and HDMECs

A comparison between cell invasion of hOBs and HDMECs was used to understand cell type-specific behaviour. Generally, osteoblasts migrated further than endothelial cells both in terms of absolute and median distance travelled. Similar to osteoblasts, endothelial cells appeared to migrate preferentially through isotropic 3D scaffolds exhibiting larger pore sizes and *d*_c_ as seen for S_1−3_, however, the effect was much less apparent than for hOBs.

The median cell position of HDMECs slightly increased in anisotropic scaffolds with increasing *d*_c_ seen for S_4−6_. Interestingly, for HDMECs seeded on both S_5_ and S_6_, a remarkably low distance of the furthest cell travelled was detected. As these samples exhibited the most alignment and smaller pore sizes at the top region than all other scaffolds, it could be hypothesized that HDMECs in monoculture tend to migrate further in scaffolds with isotropic structures and/or larger pore sizes. This could be attributed to the tendency of endothelial cells to cluster together to form the cellular junctions required for cell survival, resulting in fast occlusion of the pores due to the small and highly aligned structures [31]. In accordance with the literature, HDMECs in mono-culture were found to exhibit reduced cell migration, proliferation and cell survival compared with osteoblasts, which stresses the need for a HDMEC-conducive scaffold material in conjunction with a supportive cell type to induce the desired behaviour [29, 32]. The data generated here is fundamental to inform scaffold design for HDMEC infiltration and to establish a hOB culture throughout a 3D scaffold to stabilize future co-cultures with HDMEC.

## Conclusion

The purpose of this work was to gain insight into cell type-specific responses to 3D scaffold biochemistry and architecture. It was observed that lower cross-linking levels, up to 30% SC, supported appropriate native-like cell attachment for both hOBs and HDMECs. Scaffolds with a 30% SC cross-linking level or higher also showed appropriate stability. Scaffolds with 30% SC cross-linking, therefore, offered the best combination in terms of mechanical stiffness and cellular response. Analysis of architectural features, pore size, *d*_c_, pore homogeneity and alignment showed a time-dependent impact on the cell migration profile of both cell types, with *d*_c_ playing a more dominant role at earlier stages and pore size becoming more relevant at later stages of culture. Additionally, it was revealed that alignment plays a key role in increasing cell migration with increasing pore size and *d*_c_ having a stronger impact on aligned structures as opposed to non-aligned samples. It is hypothesized that increasing the pore size of anisotropic scaffolds would result in increased migration behaviour in comparison to isotropic counterparts having a comparable pore size. A comparison between the cell types revealed a similar impact of architectural features on the cell migration but the impact appeared to be much less apparent for HDMECs. Overall, endothelial cells exhibited the least cell migration, proliferation and survival and could benefit from future osteoblast stabilization in co-culture experiments.

## Supplementary Material

rbad015_Supplementary_Data

## Data Availability

The open access data for this article is available at https://doi.org/10.17863/CAM.93072 *Conflicts of interest statement*. None declared.
