# Supplementary material for: Adjusting the physico-chemical properties of collagen scaffolds to accommodate primary osteoblasts and endothelial cells
Source: Regen Biomater. 2023 Mar 10;10:rbad015. doi: 10.1093/rb/rbad015 (PMC10019812; doi:10.1093/rb/rbad015)
Supplement: rbad015_Supplementary_Data [file rbad015_supplementary_data.docx]

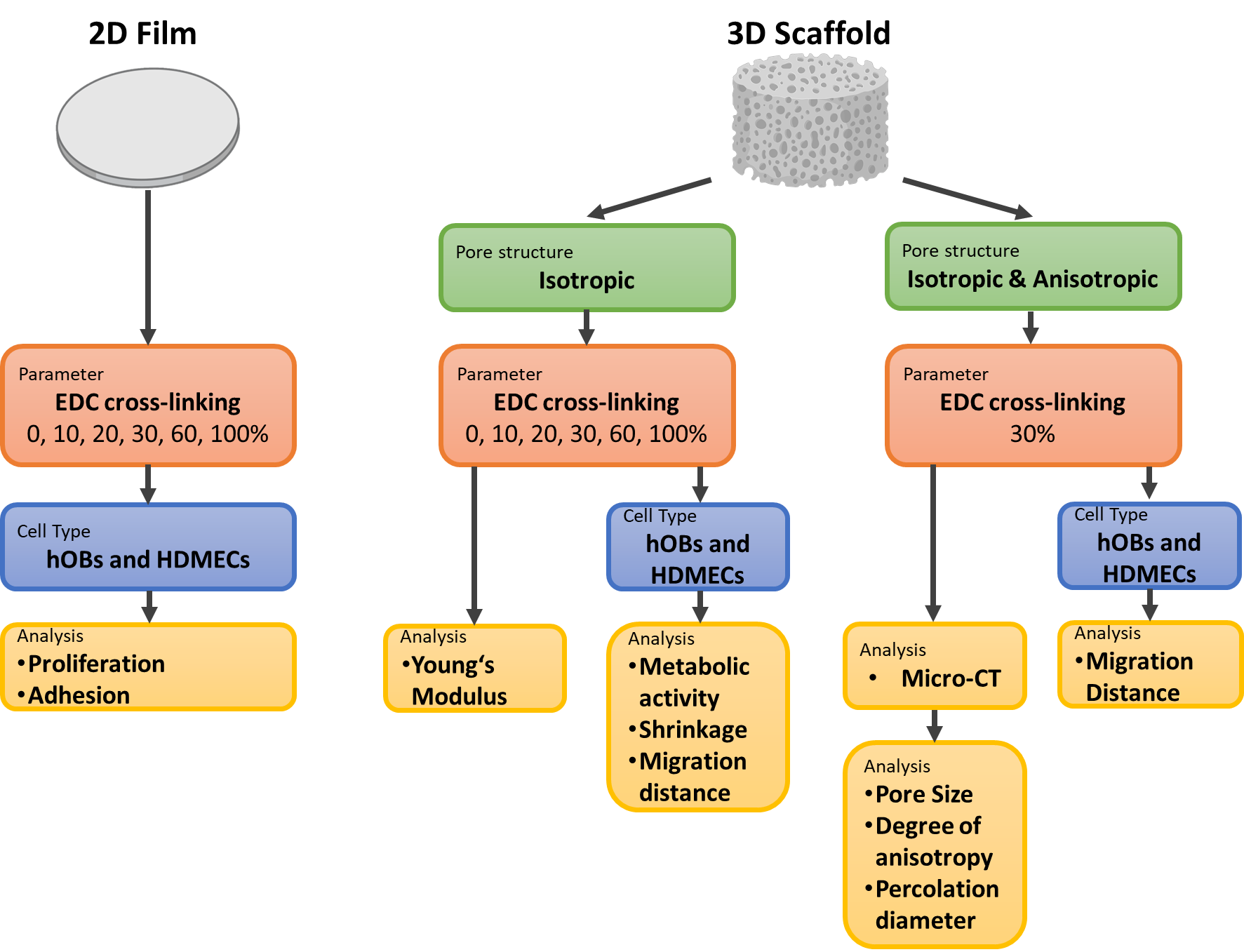


Supplementary Figure 1: Schematic overview of the material form, crosslinking density, cell types and analysis methods used.
